# Optimal Blood Pressure Keeps Our Brains Younger

**DOI:** 10.3389/fnagi.2021.694982

**Published:** 2021-10-05

**Authors:** Nicolas Cherbuin, Erin I. Walsh, Marnie Shaw, Eileen Luders, Kaarin J. Anstey, Perminder S. Sachdev, Walter P. Abhayaratna, Christian Gaser

**Affiliations:** ^1^Centre for Research on Ageing, Health and Wellbeing, Australian National University, Canberra, ACT, Australia; ^2^College of Engineering & Computer Science, Australian National University, Canberra, ACT, Australia; ^3^School of Psychology, University of Auckland, Auckland, New Zealand; ^4^Neuroscience Research Australia, Sydney, NSW, Australia; ^5^School of Psychiatry, University of New South Wales, Sydney, NSW, Australia; ^6^Centre for Healthy Brain Ageing (CHeBA), School of Psychiatry, University of New South Wales, Sydney, NSW, Australia; ^7^ANU Medical School, Australian National University, Canberra, ACT, Australia; ^8^Department of Neurology, Jena University Hospital, Jena, Germany; ^9^Department of Psychiatry and Psychotherapy, Jena University Hospital, Jena, Germany

**Keywords:** MAP—mean arterial pressure, systolic, diastolic, hypertension, machine learning, MRI

## Abstract

**Background**: Elevated blood pressure (BP) is a major health risk factor and the leading global cause of premature death. Hypertension is also a risk factor for cognitive decline and dementia. However, when elevated blood pressure starts impacting cerebral health is less clear. We addressed this gap by estimating how a validated measure of brain health relates to changes in BP over a period of 12 years.

**Methods**: Middle-age (44–46 years at baseline, *n* = 335, 52% female) and older-age (60–64 years, *n* = 351, 46% female) cognitively intact individuals underwent up to four brain scans. Brain health was assessed using a machine learning approach to produce an estimate of “observed” age (BrainAGE), which can be contrasted with chronological age. Longitudinal associations between blood pressures and BrainAGE were assessed with linear mixed-effects models.

**Results**: A progressive increase in BP was observed over the follow up (MAP = 0.8 mmHg/year, SD = 0.92; SBP = 1.41 mmHg/year, SD = 1.49; DBP = 0.61 mmHg/year, SD = 0.78). In fully adjusted models, every additional 10 mmHg increase in blood pressure (above 90 for mean, 114 for systolic, and 74 for diastolic blood pressure) was associated with a higher BrainAGE by 65.7 days for mean, and 51.1 days for systolic/diastolic blood pressure. These effects occurred across the blood pressure range and were not exclusively driven by hypertension.

**Conclusion**: Increasing blood pressure is associated with poorer brain health. Compared to a person becoming hypertensive, somebody with an ideal BP is predicted to have a brain that appears more than 6 months younger at midlife.

## Introduction

Elevated blood pressure (BP) is a major health risk factor and a leading global cause for premature death (Egan and Stevens-Fabry, 2015; Rahimi et al., 2015). In addition, hypertension is a demonstrated risk factor for dementia, and recent findings indicate a non-linear dose-response between systolic and diastolic blood pressure levels and incident dementia (Wang et al., 2018). This complex dose-response is known to be modulated by age, and by the progression of the underlying pathology, which develops over decades (e.g., amyloid plaques, neurofibrillary tangles, cerebrovascular disease). The point at which elevated blood pressure starts to impact cerebral health, and the extent of that impact, is less clear. In this study, we address this gap by estimating how a well-validated measure of brain age (BrainAGE; Franke et al., 2013; Gaser et al., 2013; Luders et al., 2016; Cole et al., 2019; Elliott et al., 2019), which reflects global brain health, relates to differences and changes in BP in community-living individuals over a follow-up of 12 years. Thus, we seek to answer the question “Does the brain of individuals with optimal blood pressure stay younger for longer?”

Worldwide, approximately 31% of all adults suffer from hypertension and a further 25–50% suffer from pre-hypertension, also referred to as phase 1 hypertension in the latest American Heart Association guidelines (Egan and Stevens-Fabry, 2015; Rahimi et al., 2015). Both hypertension and pre-hypertension are associated with an increased risk of coronary heart disease, stroke and cardiovascular disease (Huang et al., 2013; Son et al., 2018; Satoh et al., 2019). The risk increases exponentially across the diastolic and systolic blood pressure ranges above the minimum risk levels, which have been estimated at 60–74 mmHg for diastolic and 90–114 mmHg for systolic blood pressure (Rapsomaniki et al., 2014). Moreover, those suffering from pre-hypertension have a two-fold increased risk of developing hypertension (Leitschuh et al., 1991). Although hypertension is more prevalent at older ages, it is becoming increasingly common at younger ages. In the US, 7.5% of 18–39 year-olds and 33.2% of 40–59 year-olds suffer from hypertension, and substantially higher rates have been reported in some Asian countries (Son et al., 2018).

A clear link has already been established between hypertension and the development of cerebrovascular disease (Meissner, 2016). In addition to hemorrhagic strokes, elevated blood pressure is associated with cerebral micro-bleeds and with more diffuse brain changes that can be detected using Magnetic Resonance Imaging (MRI; e.g., as white matter hyperintensities, cortical thinning, enlarged Virchow-Robin spaces, brain atrophy; Alateeq et al., 2021). These changes are known to reflect pathologic microscopic processes in the underlying tissue. However, their diffuse nature makes it difficult to precisely quantify their presence and co-occurrence and therefore hampers the detection of early effects of increasing BP on brain structure.

Assumption-free machine learning approaches that consider all the information present in a brain scan without the need for *a priori* definition of regions of interest have been effectively implemented to assess the impact of several conditions on cerebral health. One such approach is the use of relevance vector machines to estimate the “brain age” of individuals based on their MRI scans. The estimated brain age can then be compared to the chronological age to determine whether specific exposures are associated with “younger-looking” or “older-looking” brains in a specific population. For example, older brain age has been detected in individuals with mild cognitive impairment (Gaser et al., 2013), with type 2 diabetes (Franke et al., 2013), exposed to maternal nutrient restriction during early gestation (Franke et al., 2018), with poor personal health markers (Franke et al., 2014), and *APOE ɛ4* carriers (Löwe et al., 2016), while younger brain age has been demonstrated in people who meditate (Luders et al., 2016), or make music (Franke and Gaser, 2019). Apart from not requiring an *a priori* determination of which brain regions should be selected for investigation in relation to a particular research question of risk factors, a major benefit of this type of approach is that it does not rely exclusively on a single index of brain integrity such as brain volume. Instead, it integrates information across key explanatory regions, which might reflect relative atrophy, vascular lesions, white matter hyperintensities, as well as other contributors which can influence MRI signals such as iron deposition, inflammation, myelination.

Using the same approach, the aim of the present study is to estimate the brain age in a large sample of people aged in their 40s to 70s for whom longitudinal MRI scans and rich epidemiological data are available and to investigate how the full range of blood pressure relates to cerebral health over time. We predicted that individuals with higher blood pressure and those suffering from hypertension would present with a higher BrainAGE. Importantly, in this research, we conceptualize BrainAGE as a marker of brain health with higher BrainAGE suggesting poorer brain health. This is because extensive research is available indicating that BrainAGE (and similar approaches)—in addition to being methodologically robust and reliable (Franke and Gaser, 2012, 2019; Baecker et al., 2021)—is associated with cognitive decline, the transition from MCI to Alzheimer’s disease, and markers of the underlying pathology and its main genetic risk factor, APOE genotype (Gaser et al., 2013; Löwe et al., 2016; Wang et al., 2019). Moreover, BrainAGE is significantly increased in several chronic conditions including type 2 diabetes (Franke et al., 2013), stroke (Egorova et al., 2019), Parkinson’s disease (Beheshti et al., 2020), Multiple Sclerosis (Cole et al., 2020), and known health and lifestyle risk factors for cardiovascular health, neurodegeneration, brain ageing, and dementia (Bittner et al., 2021).

## Materials and Methods

### Study Population

**Figure 1:**
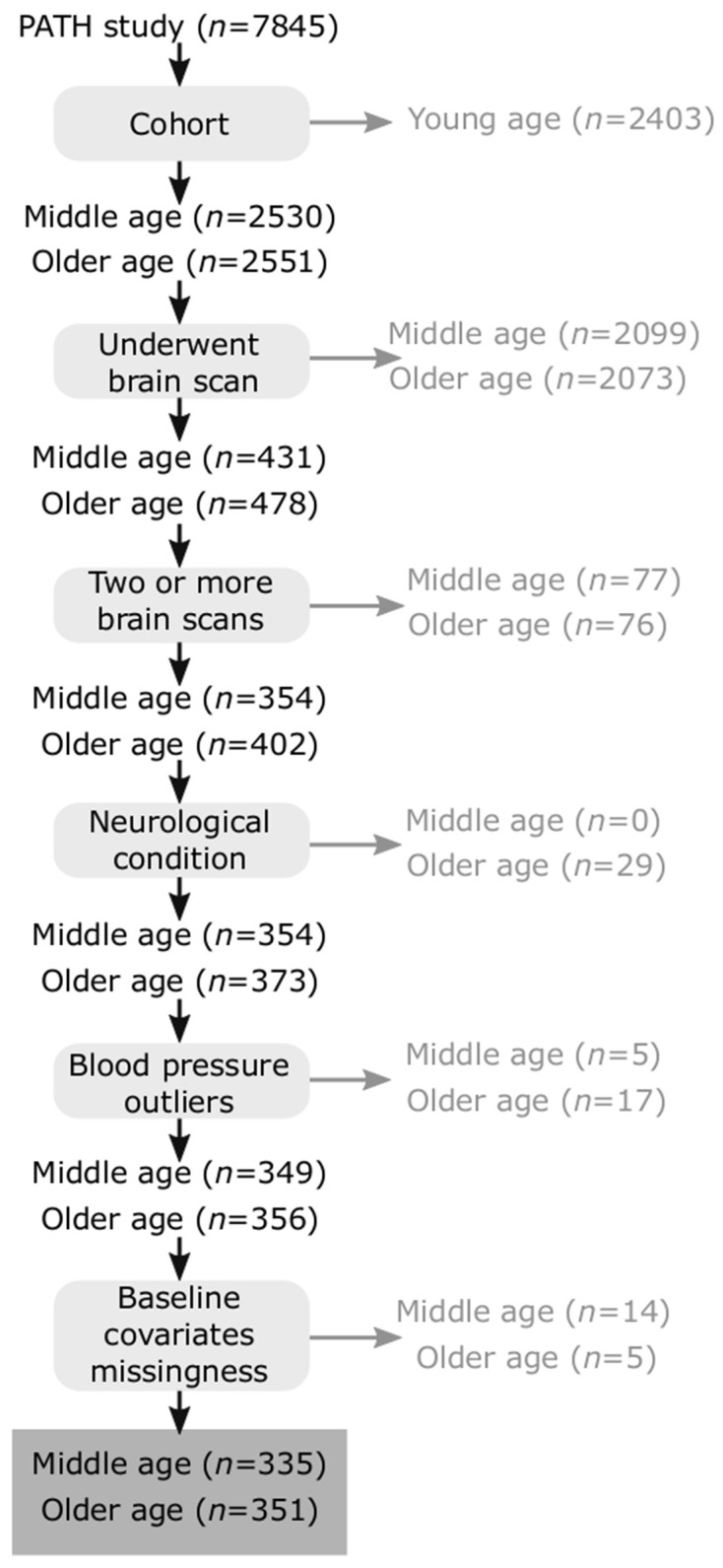
Flowchart of participants included and excluded from analyses.

Participants included in the present study were selected from the larger PATH Through Life (PATH) project which has been described elsewhere (Anstey et al., 2012). Briefly, PATH randomly sampled individuals from the electoral roll of the city of Canberra and the adjoining town of Queanbeyan across three age groups. The focus of this investigation is on the middle-age (MA; *n* = 431) and older-age (OA; *n* = 478) participants who undertook a brain scan and were aged 44–46 years and 60–64 years respectively at first MRI assessment. Participants were followed up for up to four waves of assessment over a 12 year period and were included on the basis of having two or more brain scans (MA: *n* = 354, OA: *n* = 402). Participants were excluded if they had neurological conditions (stroke, MMSE < 25, either Parkinson’s or Dementia diagnosis at any part of the study (MA: *n* = 0, OA *n* = 29) assessed based on a detailed neuropsychological assessment and consensus diagnosis using established criteria as well as self-report of a diagnosis established by a clinician (Cherbuin et al., 2019). Other inclusion criteria included blood pressure exceeding three standard deviations from the mean (MA: *n* = 5, OA: *n* = 17), or missing key covariates at baseline (Figure 1). This resulted in a final sample of 686 participants (MA: *n* = 335, 52% female; OA *n* = 351, 46% female) with 180 (26%) having two, 287 (42%) having three, and 219 (32%) having four brain scans over the follow-up. Compared with the broader PATH sample at baseline (MA; *n* = 2,530; OA: *n* = 2,551), selected participants had a slightly higher education (14.12 excluded vs. 14.39 years included, *t* = 2.45, *p* = 0.01) but were not significantly older (53.06 years excluded vs. 53.39 years included, *t* = 0.81, *p* = 0.41) and did not differ in terms of sex (*χ*^2^ = 1.08, *p* = 0.29) or intracranial volume (ICV = 1,585,868 mm^3^ excluded vs. 1,554,810 mm^3^ included, *t* = 1.51, *p* = 0.13).

### Blood Pressure

Sitting systolic and diastolic brachial blood pressure (SBP/DBP) were measured on the left upper arm at each assessment using an Omron M4 monitor after a rest of at least 5 min using a medium or large cuff as required and were computed over two measurements. Participants were classified as hypertensive if their mean systolic or diastolic blood pressure measures were higher than 140 and 90 mmHg respectively or if they took anti-hypertensive medication. Anti-hypertensive medication was assessed by self-report at each assessment. Mean arterial pressure (MAP) was calculated with the formula 1/3(SBP) + 2/3(DBP) and centered on 90. Participants were considered to have optimal BP if their DBP was <75 mmHg and their SBP was <115 mmHg (Rapsomaniki et al., 2014), which corresponds to an optimal MAP of 90 mmHg or below. These thresholds were selected based on findings from large studies indicating that SBP of 90–114 mm Hg and DBP of 60–74 mmHg were associated with the least adverse cardiovascular outcomes (Rapsomaniki et al., 2014; Li et al., 2021).

### Socio-demographic and Health Measures

Chronological age across up to three follow-up assessments was computed as baseline age in years and months plus the precise interval (years, months and days) between each assessment. Total years of education, diabetes mellitus, depression symptomatology (Goldberg depression; Goldberg et al., 1988), and smoking (ever) were assessed by self-report. Body mass index (BMI) was computed with the formula weight (kg)/height × height (m^2^) based on a self-report of weight and height. *APOE ɛ4* genotype was determined based on buccal swabs using QIAGEN DNA Blood kits (#51162; QIAGEN, Hilden, Germany). Participants were classified as *APOE ɛ4* carriers if they possessed one or two ε4 alleles. To preserve sample size across waves, the 7% or less missingness across these covariates was dealt with *via* 5,000 iterations Missing Value Analysis in SPSS.

### MRI Scan Acquisition and Image Analysis

Detailed imaging protocols are provided in the Supplementary Material (Supplementary Table 1) and are extensively published (Shaw et al., 2016a,b; Fraser et al., 2018). Briefly, at each wave, all participants were imaged with a T1 3D fast-field echo sequence on a 1.5T scanner of the same type. Some scanner/protocol changes occurred between assessments and to control for variance owing to these changes the volumetric data were orthogonalized with respect to a scanner covariate, as described elsewhere (Shaw et al., 2016a,b; Fraser et al., 2018).

### BrainAGE

As described previously (Franke et al., 2010), pre-processing of the T1-weighted images was done using the SPM8 package^1^ and the VBM8 toolbox^2^, running under MATLAB. All T1-weighted images were corrected for bias-field inhomogeneities, then spatially normalized and segmented into gray matter, white matter, and cerebrospinal fluid within the same generative model (Ashburner and Friston, 2005). The gray matter images were spatially normalized using an affine registration and smoothed with a 4-mm full-width-at-half-maximum kernel and resampled to a spatial resolution of 4 mm.

The BrainAGE framework, which has been extensively described and validated elsewhere, and used to investigate other clinical conditions (Franke et al., 2010; Luders et al., 2016; Cole et al., 2019; Franke and Gaser, 2019), was applied to the processed gray matter images. Briefly, this approach comprises three analytical steps, including data reduction, training of the algorithm, and estimation of BrainAGE. Data reduction is achieved through principal component analysis (PCA) as many MRI scan voxels are highly correlated and provide redundant information, and because using PCA has been shown to produce more sensitive measures of brain health than approaches which do not apply a data reduction step (Franke et al., 2010). The purpose of the training step, which is based on a machine learning pattern recognition method, specifically relevance vector regression (RVR; Tipping, 2001), is to identify the most accurate predictive statistical model. The BrainAGE algorithm was trained using 2,601 images from the PATH study spanning the ages of 44–76 years, since participants were randomly selected from the population in this study this sample provides the best reference for this investigation. Finally, individual BrainAGE scores are estimated by using a leave-one-out approach cross-validation. The individual BrainAGE estimate produced represents a deviation in years from chronological age. A BrainAGE of 0 means that a person’s brain appears to be the same age as their chronological age. In contrast, a negative BrainAGE indicates that a brain appears younger, and a positive BrainAGE indicates that a brain appears older than the person’s chronological age. “The Spider” package^3^, a freely available toolbox running under MATLAB, was used to train the BrainAGE estimation model as well as to predict individual brain ages. Finally, the shared variance between chronological age and the estimated brain age measure was removed using a regression approach to ensure the final BrainAge measure was not correlated with chronological age.

### Statistical Analysis

Statistical analyses were computed using the R statistical package (version 3.2). Group differences (gender and age group) were tested using Chi-square tests for categorical data and *t*-tests for continuous variables. Mixed-effects analyses were conducted to test the association between BP (MAP, DBP and SBP) and BrainAGE while controlling for age and sex (base model) as well as for education, diabetes mellitus, BMI, smoking, depression, physical activity, alcohol intake, and *APOE ɛ4* genotype (fully adjusted model). Anti-hypertensive medication effects were tested based on treatment status at each assessment. To clarify the effects of time and the effects of the cohort, age was decomposed into two variables: time in study (years from baseline) and cohort (the 40s or 60s).

## Results

Participants’ demographic measures are presented in Table 1. MA had, on average, a higher education level than OA, but had a lower DBP, SBP and also was less likely to be hypertensive, be on hypertension medications, or to have diabetes. Across the whole cohort, men had a higher education level, SBP and DBP, undertook more physical activity, and were more likely to be hypertensive than women.

**Table 1:** Participants’ demographic characteristics.

Measures	Whole sample (*n* = 686)	40s (*n* = 335)	60s (*n* = 351)	*t*/*χ*^2^ test (*p* value)	Males (*n* = 352)	Females (*n* = 334)	*t*/*χ*^2^ test (*p* value)
Age, years (SD)	55.28 (8.04)	47.18 (1.36)	63.01 (1.42)	−149.07 (<0.001)*	55.65 (8.06)	54.89 (8.00)	1.24 (0.214)
Education, years (SD)	14.49 (2.49)	14.89 (2.24)	14.11 (2.65)	4.19 (<0.001)*	14.85 (2.37)	14.11 (2.55)	3.93 (<0.001)*
Total years in study (SD)	11.51 (1.68)	11.53 (1.66)	11.50 (1.70)	0.03 (0.97)	11.34 (1.74)	11.59 (1.60)	−1.94 (0.05)
SBP, mmHg (SD)	131.80 (18.24)	125.41 (16.83)	137.90 (17.45)	−9.54 (<0.001)*	135.72 (16.58)	127.68 (19.02)	5.89 (<0.001)*
DBP, mmHg (SD)	81.70 (10.02)	80.83 (9.89)	82.53 (10.09)	−2.23 (0.026)*	84.15 (9.92)	79.13 (9.47)	6.78 (<0.001)*
MAP, mmHg (SD)	98.40 (11.83)	95.69 (11.57)	100.99 (11.51)	−6.01 (<0.001)*	101.34 (11.13)	95.31 (11.77)	6.89 (<0.001)*
BMI, kg/m^2^ (SD)	26.91 (4.43)	27.22 (4.61)	26.61 (4.23)	1.79 (0.074)	27.10 (3.82)	26.70 (4.98)	1.18 (0.240)
Physical activity, METs/day	39.15 (34.95)	37.79 (31.78)	40.44 (37.72)	−1.00 (0.319)	44.92 (37.78)	33.07 (30.58)	4.53 (<0.001)*
Smoker, *n* (%)	305 (44.46%)	154 (45.97%)	151 (43.02%)	0.49 (0.484)	138 (41.32%)	167 (47.44%)	2.36 (0.124)
Hypertension, *n* (%)	306 (44.61%)	93 (27.76%)	213 (60.68%)	73.86 (<0.001)*	117 (50.02%)	113 (33.83%)	24.92 (<0.001)*
BP Med, *n* (%)	133 (19.39%)	29 (8.66%)	104 (29.63%)	46.91 (<0.001)*	61 (18.26%)	72 (20.45%)	0.40 (0.529)
Diabetes, *n* (%)	86 (12.54%)	21 (6.27%)	65 (18.52%)	22.35 (<0.001)*	40 (11.98%)	46 (13.07%)	0.10 (0.752)
*APOE ɛ4*, *n* (%)	204 (29.74%)	101 (30.15%)	103 (29.34%)	0.02 (0.883)	94 (28.14%)	110 (31.25%)	0.65 (0.420)

Note. * indicates significance at *p* < 0.05.

### BrainAGE Characteristics

Group- and wave-specific BrainAGE are reported in Supplementary Table 2. The Pearson correlation between BrainAGE and chronological age was −0.037 (*p* = 0.09), and the mean absolute deviation of measurement between these measures was 1.26 years. Together this indicates that BrainAGE indexed brain features unrelated to chronological age. On average, BrainAGE did not differ between OA (range −13.19–16.24 years) and MA (range −11.50–16.23 years), where a lower value indicates a “younger” and a higher value an “older” appearing brain compared to chronological age. However, on average, women had a lower BrainAGE than males by almost 10 months (female mean = −0.55 years, male = 0.26 years, *p* < 0.01).

### Blood Pressure Characteristics

The mean, systolic and diastolic blood pressures (Table 1) were significantly higher in OA compared to MA (5%, 9%, 2% respectively), and in men compared to women (5.9% for all measures). While more than 28% of MA and more than 61% of OA were hypertensive, only 9% of MA and 30% of OA reported taking anti-hypertensive medication. There were 64 MA and 19 OA participants who presented with optimal BP (DBP < 75 and SBP < 115), of whom seven were on anti-hypertensive medication (MA: 2; OA: 5). A progressive increase in BP was observed over the follow-up (MAP = 0.8 mmHg/year, SD = 0.92; SBP = 1.41 mmHg/year, SD = 1.49; DBP = 0.61 mmHg/year, SD = 0.78). MA experienced a 5% greater increase in MAP and a 28% greater increase in DBP than OA, while OA experienced a 24% greater increase in SBP.

### Associations Between Blood Pressure and BrainAGE

**Figure 2:**
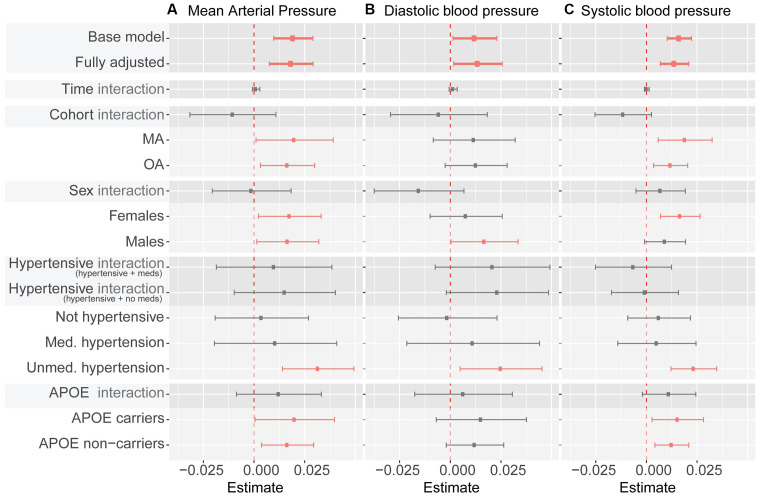
Summary of blood pressure measures as predictor of BrainAGE. Note. Whiskers indicate 95% confidence intervals. Dashed line indicates zero. “Interaction” indicates interaction term between listed predictor (cohort, sex, hypertensive category, and APOE ε4 carrier status) and blood pressure measures (mean arterial pressure in panel **A**, diastolic blood pressure in panel **B**, systolic blood pressure in panel **C**). Statistically significant coefficients are depicted in pink. All coefficients except for the base model (which controls for age and sex only) control for sex, cohort, time in study, smoking, education, physical activity, BMI, diabetes, depression, APOE ε4 carrier status, alcohol intake, hypertension, and hypertensive medication (except when subgroup analyses use a specific variable as a grouping criterion). Model coefficients can be found in Table 1, and Supplementary Tables 2–4.

Associations between BrainAGE and mean, diastolic and systolic BP are presented in Figure 2. None of the analyses revealed an interaction between BP and Time-in-Study or a random effect of blood pressure on BrainAGE indicating that a change in BrainAGE over time was not predicted by baseline BP or by a change of BP over time (Supplementary Table 2). Therefore, only fixed effects are reported below.

### MAP, SBP, and DBP

In fully adjusted models, fixed effects indicated that every 1 mmHg higher MAP over 90 was significantly associated with just under a week (6.57 days) greater BrainAGE (Figure 2A, Table 2, Supplementary Table 3) indicative of older-appearing brains.

**Table 2:** Mixed-effects model results.

	Mean arterial pressure		Systolic blood pressure		Diastolic blood pressure	
	Base model	Controlled model	Base model	Controlled model	Base model	Controlled model
Mean arterial pressure	0.019***	0.018***	0.015***	0.014***	0.016**	0.014*
	(0.009, 0.028)	(0.008, 0.029)	(0.008, 0.021)	(0.007, 0.021)	(0.005, 0.027)	(0.002, 0.026)
Time in study (years)	0.035***	0.037***	0.026**	0.029**	0.041***	0.041***
	(0.017, 0.054)	(0.017, 0.057)	(0.008, 0.044)	(0.009, 0.049)	(0.023, 0.059)	(0.020, 0.062)
Cohort (OA relative to MA)	−0.402	−0.565	−0.536	−0.667*	−0.322	−0.521
	(−0.968, 0.164)	(−1.157, 0.026)	(−1.107, 0.035)	(−1.261, −0.072)	(−0.888, 0.244)	(−1.114, 0.072)
Sex (Female relative to Male)	−0.837**	−0.775**	−0.840**	−0.788**	−0.858**	−0.800**
	(−1.402, −0.272)	(−1.357, −0.193)	(−1.405, −0.274)	(−1.370, −0.205)	(−1.423, −0.292)	(−1.381, −0.220)
*APOE ɛ4* carriers (relative to non-carriers)		−0.079		−0.085		−0.072
		(−0.693, 0.535)		(−0.701, 0.530)		(−0.686, 0.543)
Unmedicated hypertension only		−0.01		0.004		0.017
		(−0.283, 0.263)		(−0.266, 0.275)		(−0.254, 0.289)
Non-hypertensive only		−0.04		0.012		−0.106
		(−0.378, 0.299)		(−0.329, 0.354)		(−0.437, 0.224)
Constant	0.073	−0.51	0.15	−0.45	0.163	−0.308
	(−0.451, 0.598)	(− 2.722, 1.702)	(−0.368, 0.669)	(− 2.661, 1.761)	(−0.358, 0.683)	(− 2.490, 1.874)
Random effects intercept	3.633 (3.427, 3.839)	3.615 (3.394, 3.803)	3.641 (3.435, 3.847)	3.622 (3.401, 3.811)	3.636 (3.430, 3.842)	3.616 (3.397, 3.867)
Random effects residual	1.590 (1.531, 1.650)	1.593 (1.529, 1.647)	1.584 (1.526, 1.643)	1.589 (1.526, 1.644)	1.592 (1.534, 1.652)	1.595 (1.532, 1.649)
Observations	2,070	2,070	2,085	2,070	2,085	2,080
Log Likelihood	−4,860.409	−4,870.309	−4,887.345	−4,868.801	−4,893.34	−4,892.469
Akaike Inf. Crit.	9,734.818	9,784.618	9,788.691	9,781.602	9,800.679	9,826.939
Bayesian Inf. Crit.	9,774.265	9,908.595	9,828.189	9,905.579	9,840.177	9,945.381

Note. *indicates significance at *p* < 0.05, **p < 0.01, ***p < 0.001. Rounded brackets include 95% confidence intervals. Controlled models include education, diabetes mellitus, BMI, smoking, depression, physical activity, alcohol intake, and *APOE ɛ4* genotype (coefficients not reported).

In fully adjusted models, fixed effects indicated that every 1 mmHg higher SBP over 114 was significantly associated with 5.11 days older brains (Figure 2B, Table 2, Supplementary Table 4).

In fully adjusted models, fixed effects indicated that every 1 mmHg higher SBP over 74 was significantly associated with 5.11 days older brains (Figure 2C, Table 2, Supplementary Table 5).

### Optimal BP

Individuals with an optimal BP (MBP < 90, SBP < 115, DBP < 75) had a significantly lower BrainAGE than those who did not have an optimal BP (mean −0.45 vs. 0.3 years at baseline, and in mixed-effects models *b* = −0.48 95% CI [−0.843, −0.119], *p* < 0.001). However, no significant difference in BrainAGE was detected when analyses were stratified by age groups (i.e., MA and OA analyzed separately).

### Hypertension

After controlling for age and sex, the association between BP and BrainAGE did not significantly differ between those who were or were not hypertensive (MAP × hypertension status interaction *b* = 0.013 95% CI [−0.010, 0.037]). Limiting the sample to those with hypertension only, there were no significant differences in BrainAGE between those who were and were not on antihypertensive medication (mean −0.08 vs. 0.22 years, *b* = −0.042, 95% CI [−0.420, 0.336]) when considering treatment at each wave.

### Sensitivity Analyses

There were no significant interactions between MAP and sex, hypertension, or *APOE ɛ4* carrier status (Figure 2, Supplementary Tables 3–5). The significant relationship between MAP and BrainAGE remained significant in most subgroup analyses (MA only vs. OA only, women only vs. men only, *APOE ɛ4* carriers only vs. non-carriers only). In subgroup analysis based on hypertension status (not hypertensive, medicated hypertension, un-medicated hypertension) MAP continued to be positively associated with BrainAGE, but only reached significance in individuals with un-medicated hypertension. This pattern was broadly similar for DBP and SPB as predictors of BrainAGE (Figure 2, Supplementary Tables 3–5).

## Discussion

The main findings of this study were that all BP measures were associated with older BrainAGE, that these associations were stronger in men than women, and were not only detected in hypertensive individuals but across the whole BP range, with individuals with optimal blood pressure presenting with the lowest BrainAGE.

It is notable that associations were very similar between all BP measures and BrainAGE, with every 1-mmHg increase above optimal thresholds being associated with a 5–7 day increase in BrainAge. On first appearance, these effect sizes may seem trivial. However, when considered for typically observed differences in BP between individuals in good cardiovascular health compared to those who are pre-hypertensive or above, their magnitude stands out. Indeed, compared to an individual with optimal blood pressure (e.g., 110/70), an individual with pre-hypertension (e.g., 135/85) would be predicted to have a brain more than 6 months older. Although, larger age deviations (up to 6.7 years) have been detected in Alzheimer’s disease (Franke and Gaser, 2012), an average difference of 6 months has high relevance as it can serve as an additional risk marker, which if combined with other risk factors, may be predictive of premature conversion to dementia. However, more longitudinal and mechanistic evidence is required to determine the extent to which BrainAGE is a risk factor for future cognitive decline.

Importantly, these effects were not uniquely driven by some extreme cases with poorly or un-controlled hypertension because sensitivity analyses showed similar associations between BP and BrainAGE in those who were normotensive, treated hypertensive, or untreated hypertensive indicating that a consistent effect was detected across the whole BP range.

As previously reported in the literature (Luders et al., 2016), women in this cohort had a lower BrainAGE than men indicating that their brains appeared on average almost 10 months younger than those of men. The underlying reasons for this effect are not completely clear. However, it is likely that differences in cardiovascular health between men and women, which are frequently reported in the literature (Cherbuin et al., 2015), contributed substantially to this difference. Indeed, 48% more men were hypertensive compared to women in this study. Moreover, the interaction between sex and blood pressure was not significant in regression analyses, suggesting that blood pressure was the likely underlying reason for the initial sex difference.

A particularly important finding is that the association between BP (all measures) and BrainAGE was not substantially different between middle-aged and older individuals. This indicates that the negative impact of elevated blood pressure on the brain do not emerge in old age but rather progressively across the lifespan. Although the present study did not investigate young adults, the fact that associations between BP and brain are already detectable in early middle-age suggests that these effects start developing in the 30s or younger. Emerging evidence suggests that this is indeed the case. For example, Shaare and Colleagues (Schaare et al., 2019) have recently shown that moderately elevated BP (≥120/80) in 19–40 year-olds was associated with smaller gray matter volume. It is therefore imperative that greater preventative efforts be directed at this population.

### Benefits of the BrainAGE Methodology

Given the relative complexity of the BrainAGE method, one might reasonably ask whether its use is justified since other more typical methods such as regional brain volumes could perhaps be more easily used and/or explained instead. The conceptual benefits of the BrainAGE methodology—including its lack of assumption of which brain regions might be most affected and its capacity to integrate a variety of mechanisms reflecting brain integrity and not just atrophy—have already been highlighted in the introduction. In addition, the BrainAGE methodology might be particularly useful in detecting subtle, diffuse effects in young to middle-age population in which relatively low levels of atrophy and therefore low variability between individuals is observed. But perhaps as important is that BrainAGE might be easier to communicate to scientifically less informed individuals. How meaningful is it to communicate to a patient that if lifestyle modifications are not embraced now to keep their BP in a healthy range their hippocampus might shrink by an additional 1%? In contrast, being able to explain that without adequate action their brain is likely to age faster such that they may acquire a dementia diagnosis 6 months earlier than they otherwise would, might send a clearer and more potent message. This issue may be even more important in communicating with younger generations who appear to be more health-conscious but might be more responsive to more proximal health messages.

### Policy and Population Health Implication

These findings support the view that maintaining blood pressure in an optimal range (SBP < 115, DBP < 75) across the lifespan starting before mid-life (i.e., in early adulthood and before) is essential to maintain good cerebral health. The premature brain ageing associated with pre-hypertension compared to optimal blood pressure (~6 months) is likely to be associated with a very large additional burden of disease and economic costs as it is expected to directly lead to a corresponding early dementia onset, all other factors being equal.

### Limitations

This study had a number of strengths and limitations. It investigated a large longitudinal neuroimaging sample of individuals randomly drawn from the population whose age covered a period of more than three decades. BP was objectively measured, and analyses contrasted different components (SBP, DBP, MAP) while also considering the impact of clinical hypertension, anti-hypertensive medication, and variation across the whole BP range. Importantly, this study applied a state-of-the-art method to assess cerebral health without limiting* a priori* what MRI information should or could contribute to this evaluation. However, limitations included the lack of data for early adulthood, the known sub-optimal precision of brachial measurements, the possible impact of other factors not measured and accounted for in the present analyses, and the punctual nature of the assessments. In addition, while the investigation of middle-age and older-age participants was a strength, some cohort differences may have explained, at least in part, differences in findings between these groups. For example, education was significantly different between age groups. However, this difference was small in the context of a well-educated population (average >14 years of education) and fully adjusted models including education and many other covariates did not produce substantially different findings. Similarly, while the proportion of participants with higher BMI and diabetes was higher in older participants, fully controlled analyses were adjusted for these factors. Furthermore, sensitivity analyses conducted separately in middle-aged and older participants demonstrated consistent associations between BP and BrainAGE in the two age groups. Thus, it is unlikely that these differences explain the present results. Finally, BMI was computed on a self-report of weight and height and may not have been completely accurate. However, a previous study including 608 older adults has investigated the accuracy of self-reports for these measures and found that while self-report overestimated height (1.24 cm) and underestimated weight (0.55 kg) and BMI (0.56 kg/m^2^), there were strong correlations (>0.95) between measured and reported data and excellent agreement between BMI categories was observed (Ng et al., 2011).

In conclusion, the present findings show that elevated BP is associated with a relatively consistent decrease in BrainAGE across middle-age and into old age which may be indicative of worsening brain health. While much is known about the risk factors leading to elevated blood pressure and ensuing hypertension, future research is required to investigate how best to prevent exposure to these factors in early to mid-adulthood. It is also critical that such findings inform policy more effectively and are communicated widely to the population.

## Data Availability Statement

The datasets presented in this article are not readily available because data sharing is constrained by study governance and participants’ consent, however it will be made available on request for the purpose of reviewing the methodology used. Data may be made available for other purposes but this is subject to formal study data sharing processes available through the authors. Requests to access the datasets should be directed to nicolas.cherbuin@anu.edu.au.

## Ethics Statement

The study was reviewed and approved by the Australian National University Ethics Committee and the ACT Health Human Research Ethics Committee. The participants provided their written informed consent to participate in this study.

## Author Contributions

NC contributed to the design of the study and to statistical analyses, and managed all aspects of manuscript preparation and submission. EW and CG provided theoretical expertise, contributed to data preparation and statistical analyses, and contributed to writing and editing the manuscript. MS provided theoretical expertise, conducted part of the neuroimaging analyses, and contributed to writing and editing the manuscript. EL, KA, PS, and WA provided theoretical expertise and contributed to writing and editing the manuscript. All authors contributed to the article and approved the submitted version.

## Conflict of Interest

The authors declare that the research was conducted in the absence of any commercial or financial relationships that could be construed as a potential conflict of interest.

## Publisher’s Note

All claims expressed in this article are solely those of the authors and do not necessarily represent those of their affiliated organizations, or those of the publisher, the editors and the reviewers. Any product that may be evaluated in this article, or claim that may be made by its manufacturer, is not guaranteed or endorsed by the publisher.

## Footnotes

^1^ http://www.fil.ion.ucl.ac.uk/spm

^2^ http://dbm.neuro.uni-jena.de

^3^ https://people.kyb.tuebingen.mpg.de/spider
