# Supplementary material for: Optimal Blood Pressure Keeps Our Brains Younger
Source: Front Aging Neurosci. 2021 Oct 5;13:694982. doi: 10.3389/fnagi.2021.694982 (PMC8523821; doi:10.3389/fnagi.2021.694982)
Supplement: Supplementary file 1 [file Data_Sheet_1.PDF]

Supplementary Method

Table S1: Scanning parameters

	scanner	TR/TE/flip angle	slice thickness	matrix size
wave 1	Philips Gyroscan 1.5T ACS-NT	28.05ms/2.64ms/30°	2.0mm	256x256
wave 2	Philips Gyroscan 1.5T ACS-NT*	8.93ms/3.57ms/8°	1.5mm	256x256
wave 3	Siemens Avanto 1.5T scanner	1160ms/4.17ms/15°	1.0mm	256x256
wave 4	Siemens Espree 1.5T scanner	1160ms/4.24ms/15°	1.0mm	256x256

* For the first 164 participants in the 40s group, TR/TE/flip angle = 8.84 ms/3.55ms/ 8°/1.5mm

Table S2. Brain Age descriptive statistics and cohort comparisons

		MA	OA	p value
Wave 1	**Brain Age**		**(N=351)**	
	Mean (SD)	NA	-0.40 (3.65)	
	Range	NA	-9.46 - 11.73	
Wave 2	**Brain Age**	**(N=335)**	**(N=349)**	0.343
	Mean (SD)	-0.25 (3.98)	-0.54 (3.96)	
	Range	-11.72 – 16.24	-11.50 – 11.88	
Wave 3	**Brain Age**	**(N=288)**	**(N=283)**	0.664
	Mean (SD)	0.21 (4.01)	0.06 (3.92)	
	Range	-8.59 – 11.59	-11.13 – 13.81	
Wave 4	**Brain Age**	**(N=252)**	**(N=239)**	0.576
	Mean (SD)	0.23 (4.26)	0.02 (3.98)	
	Range	-13.19 – 15.30	-9.07 – 16.23	

Note. p value reports on t test comparing the age cohort. MA = Middle age, OA = Old age cohorts.

Table S3. Mean Arterial Pressure as predictor of Brain Age sensitivity analyses

	Base model	Fully controlled model	Time interaction	Cohort interaction	MA only	OA only	Sex interaction	Females only	Males only	Hypertension interaction	Not hypertensive only	Medicated hypertension	Unmedicated hypertension	APOE carrier interaction	APOE carriers only	APOE non-carriers only
Mean Arterial Pressure	0.019*** (0.009, 0.028)	0.018*** (0.008, 0.029)	0.012 (-0.003, 0.028)	0.026** (0.007, 0.044)	0.020* (0.001, 0.039)	0.017* (0.003, 0.030)	0.019* (0.005, 0.033)	0.018* (0.002, 0.033)	0.017* (0.001, 0.032)	0.008 (-0.013, 0.029)	0.003 (-0.020, 0.027)	0.009 (-0.022, 0.040)	0.031*** (0.013, 0.049)	0.015* (0.002, 0.027)	0.020* (0.0003, 0.040)	0.017* (0.004, 0.029)
Time in study (years)	0.035*** (0.017, 0.054)	0.037*** (0.017, 0.057)	0.029* (0.004, 0.054)	0.036*** (0.016, 0.057)	0.035* (0.003, 0.067)	0.038** (0.011, 0.066)	0.037*** (0.017, 0.058)	0.044** (0.014, 0.074)	0.029* (0.001, 0.057)	0.039*** (0.018, 0.059)	0.053*** (0.022, 0.084)	0.016 (-0.074, 0.106)	0.043* (0.007, 0.080)	0.037*** (0.017, 0.057)	0.046* (0.010, 0.082)	0.033** (0.008, 0.057)
Cohort (OA relative to MA)	-0.402 (-0.968, 0.164)	-0.565 (-1.157, 0.026)	-0.560 (-1.152, 0.031)	-0.481 (-1.097, 0.135)			-0.564 (-1.156, 0.029)	-0.109 (-1.008, 0.789)	-0.845* (-1.645, -0.046)	-0.547 (-1.140, 0.046)	-0.108 (-0.879, 0.662)	-1.395* (-2.660, -0.130)	-0.896* (-1.684, -0.108)	-0.563 (-1.155, 0.029)	-0.268 (-1.388, 0.852)	-0.665 (-1.369, 0.038)
Sex (Female relative to Male)	-0.837** (-1.402, -0.272)	-0.775** (-1.357, -0.193)	-0.775** (-1.356, -0.193)	-0.756* (-1.338, -0.174)	-0.984* (-1.839, -0.129)	-0.558 (-1.356, 0.241)	-0.765* (-1.371, -0.158)			-0.789** (-1.372, -0.206)	-0.904* (-1.647, -0.160)	-0.234 (-1.368, 0.900)	-0.576 (-1.324, 0.172)	-0.776** (-1.358, -0.194)	-1.309* (-2.389, -0.229)	-0.496 (-1.194, 0.203)
APOE carriers (relative to non-carriers)		-0.079 (-0.693, 0.535)	-0.081 (-0.695, 0.533)	-0.077 (-0.691, 0.536)	-0.312 (-1.210, 0.586)	0.226 (-0.614, 1.065)	-0.079 (-0.693, 0.536)	-0.520 (-1.445, 0.406)	0.265 (-0.560, 1.089)	-0.088 (-0.703, 0.527)	-0.480 (-1.256, 0.295)	0.746 (-0.452, 1.943)	0.345 (-0.436, 1.125)	-0.188 (-0.829, 0.454)		
MAP interaction with hypertension (medicated)										0.010 (-0.019, 0.039)						
MAP interaction with hypertension (unmedicated)										0.015 (-0.010, 0.040)						
Medicated hypertension only					0.282 (-0.286, 0.849)	-0.065 (-0.508, 0.378)	0.039 (-0.300, 0.377)	0.277 (-0.209, 0.764)	-0.228 (-0.702, 0.246)	-0.015 (-0.444, 0.415)				0.033 (-0.306, 0.371)	0.652* (0.037, 1.268)	-0.183 (-0.587, 0.220)
Unmedicated hypertension only		-0.010 (-0.283, 0.263)	0.016 (-0.261, 0.292)	-0.007 (-0.280, 0.266)	0.081 (-0.347, 0.509)	-0.008 (-0.366, 0.350)	0.029 (-0.246, 0.303)	-0.044 (-0.446, 0.358)	0.060 (-0.318, 0.438)	-0.103 (-0.457, 0.250)				0.023 (-0.250, 0.297)	0.177 (-0.336, 0.690)	-0.030 (-0.353, 0.292)
Non-hypertensive only		-0.040 (-0.378, 0.299)	-0.017 (-0.358, 0.324)	-0.030 (-0.369, 0.309)												
MAP interaction with time in study			0.001 (-0.001, 0.003)													
				-0.010												

MAP interaction with cohort (OA relative to MA)				(-0.032, 0.011)												
MAP interaction with sex (Female relative to male)							-0.001									
							(-0.021, 0.018)									
MAP interaction with APOE (carriers relative to non-carriers)														0.012		
														(-0.009, 0.033)		
Constant	0.073	-0.510	-0.447	-0.536	-3.273	0.304	-0.559	-1.136	-1.364	-0.560	-2.719	-1.049	-0.323	-0.559	1.210	-1.249
	(-0.451, 0.598)	(-2.722, 1.702)	(-2.661, 1.768)	(-2.747, 1.676)	(-6.797, 0.251)	(-2.378, 2.986)	(-2.741, 1.623)	(-4.013, 1.741)	(-4.530, 1.802)	(-2.738, 1.617)	(-5.940, 0.503)	(-6.284, 4.187)	(-3.535, 2.889)	(-2.735, 1.618)	(-2.710, 5.131)	(-3.867, 1.369)
Observations	2070	2070	2070	2070	864	1206	2070	997	1073	2,070	961	318	791	2070	601	1469
Log Likelihood	-4860.409	-4870.309	-4875.762	-4873.459	-2053.152	- 2817.454	-4873.991	- 2360.511	-2513.248	-4876.586	-2358.040	-831.218	-1961.040	-4873.270	-1406.414	-3460.507
Akaike Inf. Crit.	9734.818	9784.618	9797.523	9792.919	4148.304	5676.907	9793.982	4763.022	5068.497	9801.172	4756.081	1702.435	3962.081	9792.540	2854.827	6963.013
Bayesian Inf. Crit.	9774.265	9908.595	9927.135	9922.531	4248.297	5783.904	9923.594	4866.022	5173.039	9936.419	4853.440	1777.676	4055.547	9922.152	2947.198	7074.152

Note. *p<0.05; **p<0.01; ***p<0.001. Models additionally control for history of smoking, education, physical activity, BMI, diabetes, alcohol intake and depression.

Table S4. Systolic Blood Pressure as predictor of Brain Age sensitivity analyses

	Base model	Fully controlled model	Time interaction	Cohort interaction	MA only	OA only	Sex interaction	Females only	Males only	Hypertension interaction	Not hypertensive only	Medicated hypertension	Unmedicated hypertension	APOE carrier interaction	APOE carriers only	APOE non-carriers only
Systolic blood pressure	0.015***	0.014***	0.013**	0.023***	0.019**	0.012**	0.010*	0.017***	0.009	0.016*	0.006	0.004	0.023***	0.011*	0.015*	0.013**
	(0.008, 0.021)	(0.007, 0.021)	(0.003, 0.023)	(0.010, 0.035)	(0.006, 0.033)	(0.004, 0.020)	(0.001, 0.020)	(0.007, 0.027)	(-0.001, 0.019)	(0.002, 0.030)	(-0.009, 0.022)	(-0.016, 0.024)	(0.012, 0.035)	(0.003, 0.019)	(0.003, 0.028)	(0.004, 0.021)
Time in study (years)	0.026**	0.029**	0.026	0.028**	0.026	0.030*	0.028**	0.035*	0.023	0.028**	0.049**	0.011	0.028	0.029**	0.038*	0.025*
	(0.008, 0.044)	(0.009, 0.049)	(-0.001, 0.054)	(0.007, 0.048)	(-0.006, 0.059)	(0.003, 0.058)	(0.008, 0.049)	(0.005, 0.065)	(-0.005, 0.051)	(0.008, 0.049)	(0.016, 0.082)	(-0.076, 0.098)	(-0.008, 0.064)	(0.009, 0.050)	(0.002, 0.074)	(0.0003, 0.049)
Cohort (OA relative to MA)	-0.536	-0.667*	-0.667*	-0.533			-0.678*	-0.242	-0.917*	-0.674*	-0.145	-1.453*	-1.103**	-0.662*	-0.365	-0.762*
	(-1.107, 0.035)	(-1.261, -0.072)	(-1.262, -0.073)	(-1.148, 0.083)			(-1.272, -0.084)	(-1.146, 0.662)	(-1.719, -0.115)	(-1.271, -0.078)	(-0.923, 0.632)	(-2.720, -0.186)	(-1.893, -0.313)	(-1.256, -0.067)	(-1.490, 0.760)	(-1.469, -0.055)
Sex (Female relative to Male)	-0.840**	-0.788**	-0.787**	-0.753*	-0.951*	-0.584	-1.079**			-0.783**	-0.887*	-0.253	-0.661	-0.789**	-1.315*	-0.509
	(-1.405, -0.274)	(-1.370, -0.205)	(-1.369, -0.205)	(-1.336, -0.171)	(-1.806, -0.096)	(-1.384, 0.215)	(-1.722, -0.436)			(-1.366, -0.199)	(-1.631, -0.143)	(-1.385, 0.879)	(-1.410, 0.087)	(-1.371, -0.206)	(-2.397, -0.232)	(-1.207, 0.190)
APOE carriers (relative to non-carriers)		-0.085	-0.086	-0.086	-0.331	0.225	-0.079	-0.524	0.259	-0.082	-0.479	0.747		-0.253		
		(-0.701, 0.530)	(-0.701, 0.529)	(-0.700, 0.528)	(-1.229, 0.567)	(-0.616, 1.066)	(-0.693, 0.536)	(-1.451, 0.403)	(-0.566, 1.085)	(-0.697, 0.534)	(-1.254, 0.296)	(-0.456, 1.950)		(-0.900, 0.393)		
SBP interaction with hypertension (medicated)										-0.006						
										(-0.025, 0.013)						
SBP interaction with hypertension (unmedicated)										-0.001						
										(-0.017, 0.016)						
Medicated hypertension only					0.229	-0.118	-0.005	0.165	-0.213	0.121				-0.023	0.570	-0.224
					(-0.335, 0.794)	(-0.566, 0.331)	(-0.347, 0.336)	(-0.327, 0.657)	(-0.690, 0.264)	(-0.363, 0.604)				(-0.365, 0.318)	(-0.056, 1.197)	(-0.630, 0.182)
Unmedicated hypertension only		0.004	0.014	-0.005	0.018	-0.049	0.003	-0.158	0.099	-0.026				-0.015	0.127	-0.065
		(-0.266, 0.275)	(-0.264, 0.291)	(-0.276, 0.266)	(-0.406, 0.443)	(-0.410, 0.312)	(-0.271, 0.277)	(-0.565, 0.250)	(-0.275, 0.474)	(-0.396, 0.344)				(-0.289, 0.259)	(-0.385, 0.639)	(-0.388, 0.259)
Non-hypertensive only		0.012	0.022	0.016												
		(-0.329, 0.354)	(-0.326, 0.369)	(-0.325, 0.358)												
SBP interaction with time in study			0.0002													
			(-0.001, 0.001)													
SBP interaction with cohort (OA relative to MA)				-0.011												
				(-0.025, 0.003)												
							0.007									

SBP interaction with sex (Female relative to male)							(-0.005, 0.019)									
MAP interaction with APOE (carriers relative to non-carriers)														0.011		
														(-0.002, 0.024)		
Constant	0.150	-0.450	-0.438	-0.455	-3.165	0.368	-0.294	-0.965	-1.288	-0.438	-2.663	-0.939	0.022	-0.433	1.347	-1.140
	(-0.368, 0.669)	(-2.661, 1.761)	(-2.651, 1.775)	(-2.664, 1.753)	(-6.687, 0.357)	(-2.314, 3.050)	(-2.474, 1.887)	(-3.840, 1.910)	(-4.458, 1.881)	(-2.616, 1.740)	(-5.886, 0.560)	(-6.154, 4.277)	(-3.148, 3.192)	(-2.610, 1.743)	(-2.569, 5.264)	(-3.760, 1.480)
Observations	2,085	2,070	2,070	2,070	864	1,206	2,070	997	1,073	2,070	961	318	791	2,070	601	1,469
Log Likelihood	-4,887.345	-4,868.801	-4,875.267	-4,871.558	-2,051.694	-2,816.992	-4,875.780	-2,357.947	-2,514.337	-4,876.328	-2,358.184	-831.754	-1,959.907	-4,871.508	-1,406.056	-3,459.805
Akaike Inf. Crit.	9,788.691	9,781.602	9,796.534	9,789.116	4,145.389	5,675.984	9,799.561	4,757.894	5,070.675	9,800.655	4,756.367	1,703.509	3,957.815	9,789.015	2,854.111	6,961.610
Bayesian Inf. Crit.	9,828.189	9,905.579	9,926.146	9,918.728	4,245.382	5,782.980	9,934.808	4,860.893	5,175.217	9,935.902	4,853.727	1,778.750	4,046.608	9,918.627	2,946.482	7,072.749

Note. *p<0.05; **p<0.01; ***p<0.001. Models additionally control for history of smoking, education, physical activity, BMI, diabetes, alcohol intake and depression.

Table S5. Diastolic Blood Pressure as predictor of Brain Age sensitivity analyses

	Base model	Fully controlled model	Time interaction	Cohort interaction	MA only	OA only	Sex interaction	Females only	Males only	Hypertension interaction	Not hypertensive only	Medicated hypertension	Unmedicated hypertension	APOE carrier interaction	APOE carriers only	APOE non-carriers only
Diastolic blood pressure	0.016**<br>(0.005, 0.027)	0.014*<br>(0.002, 0.026)	0.005<br>(-0.013, 0.022)	0.018<br>(-0.003, 0.038)	0.012<br>(-0.009, 0.032)	0.013<br>(-0.003, 0.028)	0.021**<br>(0.005, 0.036)	0.008<br>(-0.010, 0.026)	0.017*<br>(0.0002, 0.034)	-0.003<br>(-0.024, 0.019)	-0.002<br>(-0.026, 0.023)	0.010<br>(-0.023, 0.044)	0.025*<br>(0.004, 0.045)	0.012<br>(-0.002, 0.026)	0.015<br>(-0.007, 0.038)	0.012<br>(-0.002, 0.026)
Time in study (years)	0.041***<br>(0.023, 0.059)	0.041***<br>(0.020, 0.062)	0.041***<br>(0.020, 0.061)	0.040***<br>(0.019, 0.061)	0.039*<br>(0.007, 0.070)	0.042**<br>(0.013, 0.070)	0.041***<br>(0.021, 0.062)	0.046**<br>(0.015, 0.077)	0.034*<br>(0.005, 0.063)	0.042***<br>(0.021, 0.063)	0.053***<br>(0.023, 0.084)	0.019<br>(-0.073, 0.111)	0.048*<br>(0.010, 0.087)	0.040***<br>(0.020, 0.061)	0.050**<br>(0.013, 0.086)	0.036**<br>(0.010, 0.061)
Cohort (OA relative to MA)	-0.322<br>(-0.888, 0.244)	-0.521<br>(-1.114, 0.072)	-0.508<br>(-1.102, 0.085)	-0.512<br>(-1.106, 0.082)			-0.500<br>(-1.095, 0.095)	-0.062<br>(-0.962, 0.838)	-0.790<br>(-1.593, 0.012)	-0.503<br>(-1.098, 0.091)	-0.107<br>(-0.879, 0.665)	-1.336*<br>(-2.630, -0.043)	-0.820*<br>(-1.617, -0.023)	-0.517<br>(-1.111, 0.077)	-0.224<br>(-1.345, 0.897)	-0.621<br>(-1.327, 0.085)
Sex (Female relative to Male)	-0.858**<br>(-1.423, -0.292)	-0.800**<br>(-1.381, -0.220)	-0.796**<br>(-1.378, -0.214)	-0.785**<br>(-1.368, -0.203)	-1.033*<br>(-1.889, -0.177)	-0.558<br>(-1.357, 0.240)	-0.784**<br>(-1.366, -0.201)			-0.802**<br>(-1.386, -0.219)	-0.918*<br>(-1.660, -0.175)	-0.226<br>(-1.361, 0.909)	-0.568<br>(-1.317, 0.182)	-0.792**<br>(-1.374, -0.210)	-1.329*<br>(-2.408, -0.250)	-0.512<br>(-1.211, 0.187)
APOE carriers (relative to non-carriers)		-0.072<br>(-0.686, 0.543)	-0.073<br>(-0.687, 0.541)	-0.070<br>(-0.684, 0.544)	-0.307<br>(-1.207, 0.593)	0.236<br>(-0.602, 1.074)	-0.069<br>(-0.684, 0.546)	-0.513<br>(-1.439, 0.414)	0.271<br>(-0.553, 1.095)	-0.087<br>(-0.702, 0.529)	-0.485<br>(-1.261, 0.291)	0.761<br>(-0.432, 1.955)	0.370<br>(-0.410, 1.149)	-0.077<br>(-0.691, 0.538)		
DBP interaction with hypertension (medicated)										0.021<br>(-0.008, 0.049)						
DBP interaction with hypertension (unmedicated)										0.023<br>(-0.002, 0.049)						
Medicated hypertension					0.365<br>(-0.197, 0.928)	0.011<br>(-0.424, 0.447)	0.098<br>(-0.236, 0.432)	0.362<br>(-0.119, 0.843)	-0.185<br>(-0.652, 0.281)	0.140<br>(-0.194, 0.475)				0.112<br>(-0.222, 0.446)	0.758*<br>(0.157, 1.359)	-0.122<br>(-0.521, 0.277)
Unmedicated hypertension		0.017<br>(-0.254, 0.289)	0.026<br>(-0.249, 0.301)	0.012<br>(-0.263, 0.287)	0.179<br>(-0.239, 0.597)	0.084<br>(-0.260, 0.428)	0.106<br>(-0.160, 0.371)	0.079<br>(-0.311, 0.468)	0.102<br>(-0.263, 0.467)	0.117<br>(-0.151, 0.385)				0.118<br>(-0.147, 0.383)	0.294<br>(-0.199, 0.788)	0.050<br>(-0.263, 0.362)
Non-hypertensive		-0.106<br>(-0.437, 0.224)	-0.109<br>(-0.442, 0.225)	-0.107<br>(-0.443, 0.228)												
DBP interaction with time in study			0.001<br>(-0.001, 0.003)													
DBP interaction with cohort (OA relative to MA)				-0.006<br>(-0.030, 0.018)												
							-0.015									

DBP interaction with sex (Female relative to male)							(-0.038, 0.007)									
DBP interaction with APOE (carriers relative to non-carriers)														0.007 (-0.018, 0.031)		
Constant	0.163	-0.308	-0.384	-0.426	-3.286	0.348	-0.597	-1.218	-1.351	-0.642	-2.764	-1.045	-0.067	-0.559	1.286	-1.272
	(-0.358, 0.683)	(-2.490, 1.874)	(-2.597, 1.830)	(-2.639, 1.787)	(-6.819, 0.246)	(-2.335, 3.031)	(-2.777, 1.583)	(-4.101, 1.665)	(-4.516, 1.815)	(-2.824, 1.540)	(-5.990, 0.462)	(-6.274, 4.184)	(-3.278, 3.144)	(-2.739, 1.621)	(-2.636, 5.208)	(-3.892, 1.348)
Observations	2,085	2,080	2,070	2,070	864	1,206	2,070	997	1,073	2,070	961	318	791	2,070	601	1,469
Log Likelihood	-4,893.340	-4,892.469	-4,878.183	-4,876.582	-2,054.527	-2,818.898	-4,875.836	-2,362.486	-2,513.441	-4,878.483	-2,358.017	-831.117	-1,963.972	-4,876.535	-1,407.371	-3,462.152
Akaike Inf. Crit.	9,800.679	9,826.939	9,802.365	9,799.165	4,151.053	5,679.796	9,797.673	4,766.971	5,068.881	9,804.965	4,756.035	1,702.233	3,967.944	9,799.070	2,856.742	6,966.305
Bayesian Inf. Crit.	9,840.177	9,945.381	9,931.977	9,928.777	4,251.046	5,786.793	9,927.285	4,869.971	5,173.424	9,940.213	4,853.394	1,777.474	4,061.410	9,928.682	2,949.113	7,077.444

Note. *p<0.05; **p<0.01; ***p<0.001. Models additionally control for history of smoking, education, physical activity, BMI, diabetes, alcohol intake and depression.
